# Embodiment of a virtual prosthesis through training using an EMG-based human-machine interface: Case series

**DOI:** 10.3389/fnhum.2022.870103

**Published:** 2022-08-04

**Authors:** Karina Aparecida Rodrigues, João Vitor da Silva Moreira, Daniel José Lins Leal Pinheiro, Rodrigo Lantyer Marques Dantas, Thaís Cardoso Santos, João Luiz Vieira Nepomuceno, Maria Angélica Ratier Jajah Nogueira, Esper Abrão Cavalheiro, Jean Faber

**Affiliations:** ^1^Neuroengineering and Neurocognition Laboratory, Paulista School of Medicine, Department of Neurology and Neurosurgery, Federal University of São Paulo, São Paulo, Brazil; ^2^Neuroengineering Laboratory, Department of Biomedical Engineering, Institute of Science and Technology, Federal University of São Paulo, São José dos Campos, Brazil; ^3^Center of Rehabilitation Lucy Montoro, São José dos Campos, Brazil

**Keywords:** virtual reality, amputee, prosthesis, embodiment, ownership, agency

## Abstract

Therapeutic strategies capable of inducing and enhancing prosthesis embodiment are a key point for better adaptation to and acceptance of prosthetic limbs. In this study, we developed a training protocol using an EMG-based human-machine interface (HMI) that was applied in the preprosthetic rehabilitation phase of people with amputation. This is a case series with the objective of evaluating the induction and enhancement of the embodiment of a virtual prosthesis. Six men and a woman with unilateral transfemoral traumatic amputation without previous use of prostheses participated in the study. Participants performed a training protocol with the EMG-based HMI, composed of six sessions held twice a week, each lasting 30 mins. This system consisted of myoelectric control of the movements of a virtual prosthesis immersed in a 3D virtual environment. Additionally, vibrotactile stimuli were provided on the participant’s back corresponding to the movements performed. Embodiment was investigated from the following set of measurements: skin conductance response (affective measurement), crossmodal congruency effect (spatial perception measurement), ability to control the virtual prosthesis (motor measurement), and reports before and after the training. The increase in the skin conductance response in conditions where the virtual prosthesis was threatened, recalibration of the peripersonal space perception identified by the crossmodal congruency effect, ability to control the virtual prosthesis, and participant reports consistently showed the induction and enhancement of virtual prosthesis embodiment. Therefore, this protocol using EMG-based HMI was shown to be a viable option to achieve and enhance the embodiment of a virtual prosthetic limb.

## Introduction

The concept of embodiment of an external device can be defined as “the ability to process properties of this object at the sensory, motor and/or affective levels in the same way that the properties of one’s own body parts” (De Vignemont, 2011; Makin et al., 2017), which can generate a sense of ownership and/or agency (Botvinick and Cohen, 1998). The sense of ownership refers to our ability to perceive our own body and to differentiate it from other bodies or objects using sensory information (Tsakiris et al., 2007a). The sense of agency, in contrast, is related to the perception of control of one’s own body movements and distinguishing our actions from those of other people or objects (Tsakiris et al., 2007b). In this way, embodiment can induce perception of the extension of body limits, including assistive technology devices, such as wheelchairs in people with spinal cord injury (Arnhoff and Mehl, 1963), canes in blind people (Serino et al., 2007) or even prostheses in people with amputations (Mcdonnell et al., 1989; Canzoneri et al., 2013; Petrini et al., 2019).

Recent research has revealed that the prosthesis embodiment is a key point during the rehabilitation and adaptation after amputation (Makin et al., 2017; Van Den Heiligenberg et al., 2018; Petrini et al., 2019), bringing a series of benefits: more intuitive control, facilitation of learning (Imaizumi et al., 2016; Makin et al., 2017), restoration of the perception of bodily integrity (Graczyk et al., 2018; Middleton and Ortiz-Catalan, 2020), and assisting in the treatment of phantom pain and residual limb pain (Bekrater-Bodmann et al., 2021). These aspects together make possible a better physical, psychological, and cognitive adaptation, optimizing the rehabilitation process and acceptance of the prosthetic limb.

Several studies have corroborated this concept of embodiment, showing that people with amputation can better perceive the prosthesis when it is voluntarily controlled and/or provides somatosensory feedback (Marasco et al., 2011; Raspopovic et al., 2014; Hellman et al., 2015; Wijk and Carlsson, 2015; Schiefer et al., 2017; Dietrich et al., 2018; Petrini et al., 2019). Taking this into account, an EMG-based human-machine interface (HMI) is a type of system based on voluntary control and corresponded sensory feedback. This closed loop allows gradual and consistent learning of the individual’s control ability (Lebedev and Nicolelis, 2017). Furthermore, it contains important aspects underlying the device embodiment, volition and sensory stimulation (De Vignemont, 2011; Makin et al., 2017). Thus, EMG-based HMI training provides a real-time paradigm to study the embodiment process and for use as a complementary therapeutic option.

The manner in which feedback is presented is a crucial factor for learning (Sitaram et al., 2017). An interesting option that has recently emerged is the use of virtual reality (VR). Protocols involving VR are applicable in different clinical contexts (Bohil et al., 2011; Gumma and Youssef, 2019; Qian et al., 2020), including as part of training before the use of the physical prosthesis, for people with amputations (Kluger et al., 2019). Furthermore, there is an extensive literature corroborating the embodiment of bodies, limbs, or virtual objects (Cole et al., 2009; Slater et al., 2009; Sengül et al., 2012; Shokur et al., 2016; Buck et al., 2020). Considering that the learning acquired in a VR environment is transferable to the physical environment (Bunderson, 2014; Gumma and Youssef, 2019; Kluger et al., 2019; Qian et al., 2020), the induction of virtual prosthesis embodiment could help the process of training and adaptation to the use of a physical prosthesis.

Other sensory modalities, in addition to vision, can be used to provide physiological feedback, such as touch and hearing, either isolated or integrated (Donovan et al., 2016; Shokur et al., 2016). Vibrotactile stimulation on the residual limb of people with amputations represents a natural choice, with an optimal sensory transduction since it uses reminiscent peripheral sensory paths (Ehrsson et al., 2008; Antfolk et al., 2013; D’Alonzo et al., 2015). However, due to practical or technical issues, it is not always possible to use these reminiscent areas of residual limbs, either because the residual region is too short, the surgery procedure damages a nerve fiber, or the area is not easily accessed. In these cases, it would be important to have alternative body regions that, once stimulated, would provide similar results of perception (Jones et al., 2009; Shokur et al., 2016).

In this study, we have developed an EMG-based HMI and training protocol, which aggregated previous findings in the literature that had yet to be applied and integrated in the clinical context during the rehabilitation of people with amputation: myoelectric control (Maruishi et al., 2004; Sebelius et al., 2005), VR environment (Kluger et al., 2019; Qian et al., 2020) and vibrotactile stimulation (Ehrsson et al., 2008; Antfolk et al., 2013; D’Alonzo et al., 2015).

The EMG-based HMI was designed in a way that the participants could control the movements of a prosthesis immersed in a VR environment using the myoelectric activity of the residual limb, while receiving non-invasive vibrotactile stimuli applied on their back, which were mapped to represent the movements of the virtual prosthesis. The training was applied during the preprosthetic rehabilitation phase of people with transfemoral amputation. The hypothesis was that training with this EMG-based HMI, could induce and enhance virtual prosthesis embodiment.

## Case description

This is a case series study that assessed the induction and enhancement of virtual prosthesis embodiment through a training protocol with an EMG-based HMI. Case Reports Guidelines were used to develop this work. The research protocol was approved by the Ethics and Research Committee of the Universidade Federal de São Paulo (n° 3.030.942) and of the Hospital Municipal José de Carvalho Florence (n° 3.273.170), Brazil.

### Characterization of participants

For the inclusion of participants in the research, the following criteria were adopted: people with unilateral transfemoral amputation, both sexes, age between 18 and 46 years, and without previous use of prostheses. People who had open skin lesions on the residual limb or back, uncorrected visual impairment or associated neurological diseases were excluded from participation in the study. The participants provided written consent prior to the start of the study, and all ethical recommendations were followed.

Sociodemographic, physical, functional, cognitive, and psychological assessments of all participants were carried out to provide a general characterization (Table 1). Qualitative reports are provided in Supplementary Table 1 in the Supplementary Material.

**TABLE 1:** Characterization of the sociodemographic, physical, functional, cognitive, and psychological aspects of the participants.

Measures	Participants						
	A	B	C	D	E	F	G
**Sociodemographic**							
Age (years)	46	32	22	32	24	46	41
Sex	Male	Male	Male	Male	Male	Female	Male
Education (years)	11	11	11	7	5	11	5
**Physical and functional**							
Height (m)	1.69	1.87	1.81	1.79	1.69	1.67	1.75
Body mass (kg)	80.9	64.5	60.0	88.3	46.0	78.7	67.0
Amputation time (months)	11	3	11	74	21	13	5
Amputation side	Right	Right	Left	Right	Left	Right	Right
Residual limb length (cm)*^1^	34	35	37	13	20	37	30
Residual limb pain intensity*^2^	8	3	0	0	0	0	0
Phantom limb sensation	No	Yes	Yes	No	Yes	Yes	No
Functional level*^3^	34	34	37	37	39	27	30
Physical activity level*^4^	High level	Low level	High level	High level	High level	High level	High level
Residual limb hip muscle strength (kg/F)^*5^							
Flexors	29.2	17.5	14.2	16.6	14.7	15.7	17.0
Extenders	20.5	9.6	13.9	12.8	12.9	14.1	12.8
Abductors	14.8	11.4	12.2	17.2	11.3	13.7	12.4
Adductors	15.0	10.1	12.2	–^**^	9.9	11.3	10.8
**Cognitive and psychological**							
Cognitive level^*6^	26	28	23	23	20	29	18
Depression level^*7^	5	2	4	8	1	7	1
Anxiety level^*7^	4	5	7	6	2	1	3

*1 Residual limb measurement reference was made considering the distance from the greater trochanter of the femur to the distal extremity (Pedrinelli, 2004).

*2 Numerical pain scale, where ‘0’ indicates no pain and ‘10’ indicates the worst pain (Jensen et al., 1986; Hawker et al., 2011).

*3 The Amputee Mobility Predictor No Prosthesis (AMPnoPRO) assesses mobility aspects of amputees and predicts functional levels related to the use of prostheses (Gailey et al., 2002).

*4 The International Physical Activity Questionnaire - short version (IPAQ) was used to assess the level of physical activity (Matsudo et al., 2001).

*5 Measurement made using a digital dynamometer. The point of force application was considered the midpoint of the residual limb length. Three isometric contractions were performed for each muscle group, and the mean peak strength was calculated over the last 5 s of contraction (Mentiplay et al., 2015).

*6 The Montreal Cognitive Assessment (MoCA) was used to assess cognitive functions (Sarmento, 2009).

*7 The Hospital Anxiety and Depression (HAD) Scale was used to assess levels of anxiety and depression (Botega et al., 1995).

^**^ For participant “D”, it was not possible to assess the strength of the adductor muscles due to the small size of the residual limb.

**Participant A:** The limb was amputated on July 20, 2018, because of an accident involving a motorcycle and a truck. Due to trauma, there were multiple fractures and local infection, culminating in amputation.

**Participant B:** The patient had a history of an accident involving a car and a motorcycle in the year of 2011, resulting in a lower limb injury. He was bedridden for approximately 2 years and used an external fixator for fracture treatment. In December 2018 arthrodesis of the knee was performed. However, due to the complications from osteomyelitis, amputation was performed on May 7, 2019.

**Participant C:** The lower limb was amputated immediately after an accident involving a motorcycle and a truck on August 31, 2018.

**Participant D:** Amputation was performed on June 13, 2013, due to extensive injury to the lower limb after an accident involving a motorcycle and a car. The patient also sustained a right forearm fracture during that same event.

**Participant E:** Traumatic amputations occurred during an accident involving a motorcycle and a car on January 5, 2018, transfemoral in the lower limb and transradial in the upper limb left.

**Participant F:** Surgical amputation was performed on September 18, 2018, after the patient was hit by a vehicle, resulting in crushing of the limb and vascular complications.

**Participant G:** Amputation was performed on August 14, 2019, because of an accident between two motorcycles. In addition to the amputation, the patient had a fracture of the left femur that required a surgery for stabilization.

### Intervention

#### EMG-based human-machine interface

The EMG-based HMI was designed to work using the electrophysiological activity of the muscle on the residual limb. Through a real-time recording and processing of this activity, the participants were able to control the knee movements of a virtual prosthesis while receiving patterns of vibrotactile stimulation on their back, representing the current position of the virtual prosthesis.

#### Recording of muscle activity

**FIGURE 1:**
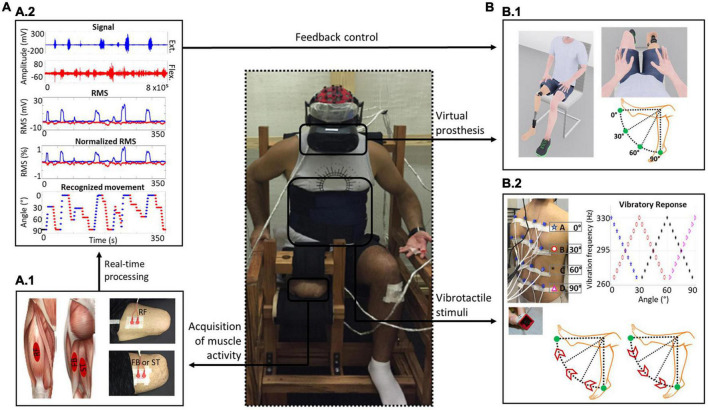
EMG-based human-machine interface scheme. **(A)** Muscle activity recording through a surface EMG. **(A.1)** Illustration of the rectus femoris (RF) (hip flexor and knee extensor), femoral biceps long head (FB) and semitendinosus (ST) muscles (hip extensors and knee flexors) and positions of the surface electrodes on these muscles responsible for controlling the movements of the virtual prosthesis knee. **(A.2)** Schematic diagram of the real-time processing of electromyographic activity and root mean square (RMS) calculations to estimate the level of muscle contraction. The RMS was normalized to the maximum voluntary isometric contraction (MVIC) of each muscle. Regarding recognition of the movement direction, the activity of the agonist muscle should be twice as high as the average of the baseline signal, and the antagonist muscle could not exceed a threshold relative to the agonist, which was initially set at 80%. The recognized EMG patterns were mapped into visual and vibrotactile feedback. **(B)** Feedback. **(B.1)** Visual feedback. Avatar modeled with a transfemoral prosthesis and visualization from the first-person perspective are shown. The range of motion available to the prosthetic knee was set between 0° and 90°. **(B.2)** Vibrotactile feedback scheme. The positioning of vibrotactile actuators on the participant’s back was organized in a 4 × 4 matrix. The paradigm for the applied vibratory stimuli was associated with the movements of the virtual prosthesis: upward vibration during knee extension and downward vibration during knee flexion. The vibratory intensity peak of a given row corresponded to a specific angle of knee movement (row A, 0°; B, 30°; C, 60°; and D, 90°), with an overlap of 30° between adjacent rows.

The activity of the rectus femoris (hip flexor and knee extensor) and femoral biceps long head or semitendinosus muscles (hip extensors and knee flexors) (Kendall et al., 1995) on the residual limb was recorded using surface electromyography (EMG). Electrode placement for each muscle and participant was determined by applying excitomotor electrical current stimulation and visualizing the muscle contraction response. These positions were mapped for each person and used in all training sessions. Two channels of an Intan Technologies^®^ chip were used to amplify the electrophysiological signals, and the chip was connected to the OpenEphys^®^ analog-digital converter board in communication with its software (Black et al., 2017; Siegle et al., 2017). The electrophysiological signals were sampled at a rate of 10 kHz (Figure 1A.1).

#### Real-time processing

Data were processed using MATLAB^®^ (R2017b). For real-time control, every 200 ms, the EMG signals in each channel were loaded in blocks of 5120 samples, resulting in a 60% overlap with the previous sample (Moreira et al., 2021). The samples were filtered using a twentieth-order IIR bandpass filter in the frequency range from 10 to 500 Hz and filtered at 60 (±2) Hz with its harmonics (Stegeman and Hermens, 2007). Then, the EMG signal in each window was resampled to 2 kHz, and its root mean square (RMS) was calculated to estimate the muscle contraction level (Staudenmann et al., 2010).

To control the feedback, two criteria needed to be satisfied: (a) Agonist muscle activation threshold. The RMS of the agonist muscle signals had to be greater than 2 standard deviations (SD) in relation to the baseline signal for the system to recognize the direction of movement (knee extension or flexion). (b) Tolerance of antagonist muscle contraction. Initially, the RMS of the antagonist muscle could not exceed 80% in relation to the agonist muscle (this parametrization was also used as a criterion for the progression in difficulty levels during training). Therefore, a higher level of EMG activity associated with the hip flexor muscle resulted in the knee extension movement of the virtual prosthesis and, simultaneously, in an upward vibrotactile stimulation pattern on the subject’s back. A higher level of EMG activity associated with the hip extensor muscle resulted in knee flexion of the virtual prosthesis and, simultaneously, in a downward vibrotactile stimulation pattern on the subject’s back (Figure 1A.2). For more details about the definition of the vibrotactile stimulation pattern, see Supplementary Figure 1 in the Supplementary Material.

#### Virtual reality environment

The virtual environment was developed on the Unity3D^®^ platform (2018.4). The environment was conceived to simulate a regular clinical room where the users could see themselves in a first-person perspective as a humanoid avatar using a transfemoral prosthesis in the corresponded lower limb. The subjects were able to control the knee extension and flexion movements of the prosthetic limb within a range between 0° and 90° (Figure 1B.1). Moreover, the virtual environment was designed to enable gamification of the protocol with different stages and motivational messages to reinforce learning. The participants accessed the virtual environment using a Samsung^®^ Odyssey Oculus Head-Mounted Display that provided a first-person view in a fixed sitting position (Slater et al., 2009, 2010) and the ability to visually explore the whole 3D virtual environment. For more details about VR environment see Supplementary Material (Supplementary Table 2).

#### Vibrotactile stimulation device

A total of 16 vibrotactile actuators (10 mm × 6 mm; 5 V-DC) were assembled in a 4 × 4 matrix and positioned on the subject’s back (Jones et al., 2009), with an average distance of 6 cm among them. Vibrotactile stimulation was applied at frequencies between 260 and 330 Hz, which is optimal for stimulating Pacinian corpuscles, the main skin vibration receptors (Kandel et al., 2014). Vibrotactile actuators were arranged in groups of 4 (organized by rows on the back), and each group was activated together (actuator activation was performed through an Arduino^®^ platform communicating in real time with MATLAB^®^ (R2017b). All actuators placed along the same row vibrated with the same intensity, with maximum intensity when the virtual prosthesis was positioned at a specific movement angle (0°, 30°, 60°, or 90°); there was a vibratory overlap of 30° with the adjacent rows to produce a continuity effect on vibratory perception (Moreira et al., 2021; Figure 1B.2). For more details on the vibrotactile stimulation device see Supplementary Table 3 and Supplementary Figure 2 in the Supplementary Material.

#### Training protocol

Two preliminary sessions were conducted prior to the start of the training protocol to familiarize participants with the EMG-based HMI. In these sessions, the participants learned to associate the residual limb muscular contraction with virtual prosthesis movements (for details, see Supplementary Figure 3 in the in the Supplementary Material). After this stage, the training was based on an operant conditioning paradigm, in which there was a progressive increase in the difficulty of the tasks with contingent feedback and rewards to reinforce learning. Overall, contingent feedback itself has a positively reinforcing effect, but this was supplemented with motivational messages, such as “congratulations,” at the end of each task block (Skinner, 1938; Kandel et al., 2014).

**FIGURE 2:**
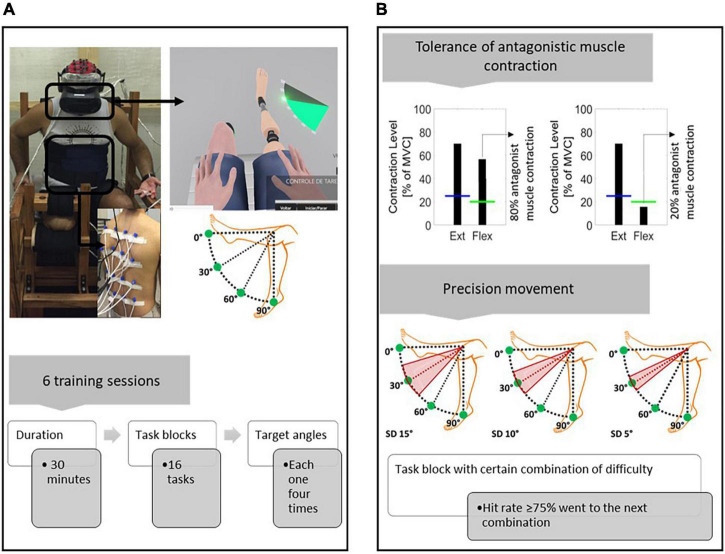
Training protocol with the EMG-based HMI scheme. **(A)** Training protocol diagram. Feedback within the virtual environment consisted of visual clues indicating the target angles that the participants had to reproduce. The target angles used were 0°, 30°, 60°, and 90°. Each angle was randomly presented four times during each task block (the participant had 20 s to establish each target angle). In addition to visual feedback, the participants received concomitant vibrotactile feedback on their back. The training sessions lasted 30 mins, and within that time, as many task blocks as possible were performed. **(B)** Difficulty of progression. Two criteria were adopted to increase the difficulty: (i) Tolerance of antagonist muscle contraction. Initially, the antagonist muscle could have up to 80% activation in comparison to the agonist muscle. The tolerance decreased progressively by 10% at each new difficulty level (the lower the tolerance was, the greater the need to isolate the agonist muscle contraction). (ii) Precision movement. To evaluate whether a target angle has been reached, different ranges of prosthesis position, in relation to the target angle, were adopted (15°, 10°, and 5°: the lower the range was, the greater the necessary precision of movement). Given a tolerance of antagonist muscle contraction, the different precision difficulties were progressively combined. If the participant had a success rate ≥ 75% on a task block with a certain combination of difficulties, the next block instituted a new combination of difficulties.

In total, six training sessions lasting 30 mins each, consisting of task blocks involving motor control were conducted twice a week. The maximum of task blocks was performed within the 30 mins. For each task, the participants moved the virtual prosthesis until they reached a specific predefined position set at four target angles: 0°, 30°, 60° or 90° (a combination of angles with targets at 0°, 45°, and 90° was also used as a preliminary stage for each new level of difficulty). To guide the movements in real time, the participants were presented with a visual clue (semicircular ruler) indicating the position to which they should move the virtual prosthesis (Figure 2A).

The following criteria were adopted to increase the task difficulty: (a) Tolerance of antagonist muscle contraction. Antagonist muscle activation up to 80% in relation to agonist was initially established, which decreased by 10% at each new difficulty level; (b) Precision of movement. For a task to be considered correctly performed, a range of positions was adopted in relation to the target angle. The difficulty levels varied from the target angle as follows: ±15°, ±10°, and ± 5°. Therefore, initially, there was no need for refined muscle control (regarding the isolation of agonist muscle contraction) and movement precision. However, this became necessary as the difficulty gradually increased (Figure 2B).

In this manner, given a particular difficulty combination (tolerance of antagonist muscle contraction and precision of movement), the participants performed a preliminary block and then a task block composed of a set of target angles, 0°, 30°, 60°, and 90° (each presented randomly four times), for a total of sixteen tasks for each block. After an attempt of 20 s, or if the target angle was hit, the next task was presented (if the participant did not hit the task within 20 s, it was considered a failure, although the participant did not receive any messages indicating the failure). The performance was assessed at the end of the task block, and the difficulty was increased if the participant had a success rate of 75% (this cutoff was heuristically calculated from previous pilot studies) or more; otherwise, the same difficulty combination was performed again.

### Embodiment assessment

We assessed a set of measurements to examine the induction and enhancement of virtual prosthesis embodiment. This test set was selected based on affective, spatial perception, and motor mechanisms. These three features were proposed by De Vignemont (2011) and underlie the development of the object’s embodiment. In addition, we investigated self-perception regarding the sense of ownership and agency. Affective, spatial perception and self-perception measurements were assessed at the beginning and end of the experimental protocol. Motor measurement was performed in all training sessions.

#### Affective measurement

**FIGURE 3:**
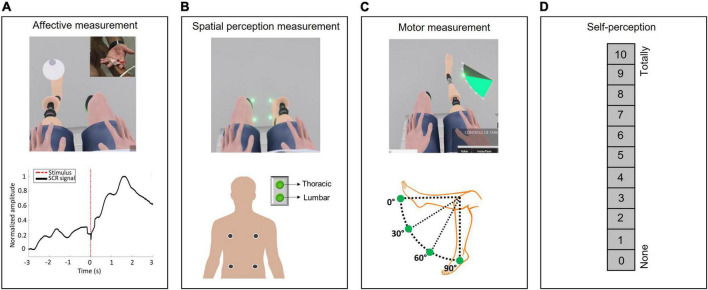
Embodiment assessment. **(A)** Affective measurement–Skin conductance response. Two surface electrodes were placed on the intermediate phalanges of the second and third left hand fingers, and the SCR was recorded once a chandelier dropped on the virtual prosthesis, representing a threatening stimulus. **(B)** Spatial perception measurement–Crossmodal congruency task (CCT). During the CCT, visual stimuli were applied within the VR environment close to the avatar’s feet (close to the hallux or heel) soon after the appearance of the visual distractor, and a vibratory stimulus was applied on the participant’s back (thoracic or lumbar). The CCT was composed of sixteen different combinations of visual and vibrotactile stimuli, each presented four times at random, for a total of sixty-four trials. The participants were instructed to press a button corresponding to the location on their back where they received the vibratory stimulation as quickly as possible while ignoring the visual distractor. **(C)** Motor measurement. The participants moved the virtual prosthesis until they reached a specific predetermined position set at four target angles: 0°, 30°, 60°, or 90°. The participants’ performances, execution time and success rates during the training were used to assess their ability to control the virtual prosthesis. **(D)** Self-perception. The participants quantified on a scale from 0 to 10, where 0 indicated “none” and 10 indicated “totally,” how much they felt the virtual prosthesis was part of their own body and how much they felt that they could control it.

Skin conductance response (SCR) was used to detect inherent physiological responses when the virtual prosthesis was threatened (Critchley, 2002; Alimardani et al., 2016). SCR acquisition was accomplished using the e-Health^®^ (2.0) system coupled to an Arduino Uno^®^, with a sampling rate of 20 Hz. The SCR recording was performed at the initial session and at the penultimate training session; for this, surface electrodes (Ag/AgCl) were placed on the intermediate phalanx of the second and third left hand fingers (Ehrsson et al., 2008). This recording was made 2 mins before and during the simulation of a threat−a chandelier falling on the virtual prosthesis (Yuan and Steed, 2010). At the beginning of the training sessions, all participants watched a video showing the fall of the chandelier on the virtual prosthesis, and they were informed that at some point during the sessions, the same event could occur, thereby minimizing the effects of surprise on the measurements (Alimardani et al., 2016). The participants did not know on which day this test would be conducted. Finally, the magnitude of the SCR was analyzed (Braithwaite et al., 2015; Figure 3A).

#### Spatial perception measurement

A crossmodal congruency task (CCT) involving a visual stimulus (visual distractors on the virtual body) and a concomitant tactile stimulus (vibratory stimulation of the participant’s back) and the respective crossmodal congruency effect (CCE) were used to identify visuotactile interference in the peripersonal space (Sengül et al., 2012; Marini et al., 2014; Shokur et al., 2016). To perform this task, the participants visualized the lower limbs of the avatar and a luminous point (visual distractor) in four different positions: on either side of the hallux or heel. In addition, four vibrotactile actuators were positioned on the participants’ backs: two were placed in the thoracic region, and two were placed in the lumbar region on both sides. Therefore, there were 16 possible stimulus combinations: 4 positions of the visual distractors and 4 positions related to the vibratory stimuli, and each combination was randomly presented four times for a total of 64 repetitions in each task block. A visual distractor was presented and followed 100 ms later by vibrating stimulation for 350 ms. The participants were then instructed to press a button based on the place on their back that they had received the vibratory stimulation while ignoring the visual distractor. They had two options: upper (thoracic) or lower (lumbar). If the participant did not press the button within 2 s, the next combination was presented. The CCT protocol consisted of observing the virtual prosthesis performing knee flexion and extension movement (at an angular speed of 45°/s for 1 min) with or without concomitant vibratory stimulation related to virtual prosthesis movements. This observation sequence was random, and the CCT task block was performed after each paradigm. All participants previously underwent training and started this task only after reaching an accuracy of 85% in localizing the vibratory stimulus. In this manner, the CCE was calculated as the difference in the reaction time between incongruent (for instance, when a visual distractor was localized on the upper part of the foot and the vibratory stimulation was in the lumbar region) and congruent conditions (for instance, when a visual distractor was localized on the upper part of the foot and the vibratory stimulation was in the thoracic region) (Maravita et al., 2003; Shokur et al., 2016; Figure 3B and Supplementary Figure 4 in the Supplementary Material).

#### Motor measurement

The participants’ performances, execution time and success rates, during the training were used to assess their ability to control the virtual prosthesis, considering the different levels of difficulty during the tasks, the tolerance for antagonist muscle contraction and the precision of movement (Figure 3C).

#### Self-perception

The participants quantified on a scale from 0 to 10, where 0 indicated “none” and 10 indicated “totally,” how much they felt the virtual prosthesis was part of their own body and how much they felt that they could control it (Armel and Ramachandran, 2003; Figure 3D).

### Data analysis

The data analyses and electrophysiological signal analyses were performed in MATLAB^®^ (R2017b). Parametric or non-parametric hypothesis tests were used based on the classification of the Kolmogorov-Smirnov test (Mohd Razali and Bee Wah, 2011). Differences were considered significant when *p <* α, where α = 0.05.

#### Affective measurement

To compare SCR magnitudes among the 4 different periods (before and after the threat, at the beginning and end of the experimental protocol) a two-way ANOVA was used with a Tukey-Kramer *post hoc* correction. A one-way MANOVA was applied followed by canonical discriminant analysis to determine whether the set of variables (SCR amplitude waveforms) exhibited specific clusters based on each period of threat exposure. The SCR signal analysis, the following steps were performed: (a) smoothing the original x(t) signal by averaging it over a 3-s sliding window with 50% overlap along the whole signal and producing a x′(t) signal; (b) calculating the phase signal from the difference y(t) = x(t)−x′(t); and (c) applying a logarithmic scale over the magnitude of the signals and considering the 3 s of signal before and 3 s after the application of the visual stimulus (i.e., the moment when the chandelier enters the visual field of the participant within the VR environment) (Braithwaite et al., 2015). The SCR signals from participant “B” were excluded from the analysis due to noise issues during registration.

#### Spatial perception measurement

Statistical comparisons were performed between CCE averages considering that the visual and tactile stimuli were applied on the same and the opposite side. Thus, a two-way ANOVA with Tukey-Kramer *post hoc* correction was applied for this comparison. The CCE calculations, the only data that were included were from correct executions, while times greater than 1500 ms and less than 200 ms were excluded (3.4% of all trials) (Sengül et al., 2012). The prior visualization of the virtual prosthesis movements with and without associated vibrotactile stimulation were both considered statistical factors at the beginning and end of the protocol.

To evaluate the relationships between CCE and SCR measures, Pearson’s correlation coefficients were calculated together with the description of the respective linear regressions for each variable pair. Only the coefficients with *p <* α, tested with a *t*-test, were considered significant.

#### Motor measurement

The following variables were analyzed: (a) execution time and (b) success rate. The target angles were separated into intermediate (30° and 60°) and extreme (0° and 90°) angles. In addition, the data were grouped into three difficulty levels related to the precision of movements, i.e., 15°, 10°, and 5° to the target angle. The task execution times were compared among these three difficulty levels while separately considering the intermediate and extreme target angles (Kruskal−Wallis test followed by Tukey-Kramer *post hoc* correction). Comparisons among execution times, while considering the intermediate and extreme target angles, were also performed (using the Mann−Whitney test), as well as the analysis of success rates by comparing among average proportions and confidence interval (CI) (95%).

#### Self-perception

Presentation of the absolute values reported by each participant (from 0 to 10) at the beginning and at the end of the protocol for the ownership and agency sense.

## Results

Virtual prosthesis embodiment and enhancement through the training protocol using EMG-based HMI was consistently observed in different analyses.

### Affective measurement

**FIGURE 4:**
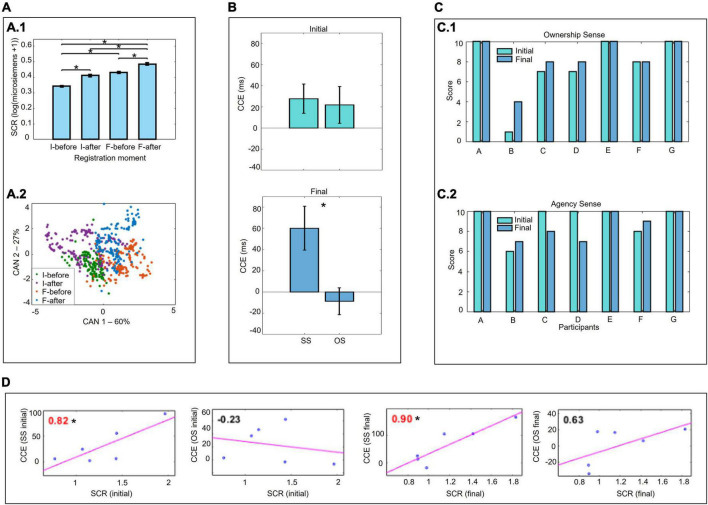
Affective, spatial perception measurements, and self-perception. **(A)** Skin conductance response (SCR) to a threat to the virtual prosthesis. **(A.1)** Two-way ANOVA with Tukey-Kramer correction. **(A.2)** Application of one-way MANOVA followed by canonical discriminant analysis. **(B)** Crossmodal congruency task (CCT) and crossmodal congruency effect (CCE) (two-way ANOVA with Tukey-Kramer correction). Comparison for stimuli applied on the same side (SS) and opposite side (OS). **(C)** Self-perception (absolute values quantified by the participants). **(C.1)** Sense of ownership. **(C.2)** Sense of agency. **(D)** Correlations between the SCR and CCE results (Pearson’s correlation coefficients). *p < 0.05.

All participants reacted affectively to a threat to the virtual prosthesis (inside the virtual environment, a chandelier fall over the prosthesis). The affective response was indicated by a significant increase in SCR after the threatening event (*F* = 53.3, *p <* 0.001), both at the beginning (*post hoc p <* 0.001−before and after the threat) and at the end of the experimental protocol (*post hoc p <* 0.001−before and after the threat). At the end with greater magnitude compared to the beginning (*F* = 85.15, *p <* 0.001; *post hoc p <* 0.001−before the threat at the end and beginning; after the threat at the end and beginning) (Figure 4A).

### Spatial perception measurement

There was a significant increase in CCE for stimuli applied to the same side of the body compared with stimuli applied to opposite sides, at the end of the training (*F* = 7.7, *p* = 0.010, *post hoc* for the preview statistical factors VR only and VR-VT *p* = 0.046). This indicated, that at the end of training, the visual stimuli applied in the virtual environment were considered close to the real body. No difference in stimulus application between sides was found in CCE at the beginning (*F* = 0.06, *p* = 0.798) (Figure 4B).

Furthermore, there was a significant correlation between the mean SCR and CCE values (stimuli applied on the same side) at the beginning and end of the training (beginning *r* = 0.82, *p* = 0.047; final *r* = 0.90, *p* = 0.014) (Figure 4D).

### Motor measurement

**FIGURE 5:**
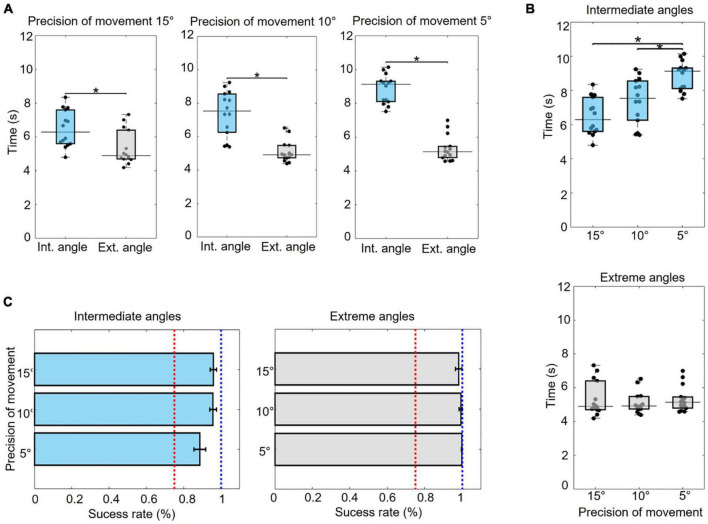
Motor measurement. **(A)** Comparison of execution times between the intermediate (30° and 60°) and extreme (0° and 90°) target angles (Mann–Whitney test). **(B)** Comparison of execution times across levels of difficulty related to the precision of movement (15°, 10°, and 5° of variation in relation to the target angle) for the intermediate and extreme angles (Kruskal–Wallis with Tukey–Kramer correction). **(C)** Success rate on tasks involving intermediate and extreme target angles at each level of required movement precision (average of the proportion and CI). The red line indicates a success rate of 75%, and the blue line indicates 100%. *p < 0.05.

Motor training with the EMG-based HMI provided an improvement in the ability to control the virtual prosthesis, considering that there was a success rate > 75%, even with the progressive increase in the difficulty of the tasks (Figure 5C). However, although the success rate was always high, the execution time was longer in the more difficult/complex conditions. With intermediate angles (30° and 60°), the time to execute the tasks was longer than with extreme target angles (0° and 90°) regardless of the precision of movement required during the task (precision of movement 15°: *U* = 263, *p* = 0.006; 10°: *U* = 288, *p <* 0.001; 5°: *U* = 301, *p <* 0.001) (Figure 5A). The difficulty associated with greater movement precision (5° range in relation to the target angle) also demanded significantly more time for task execution (H = 18.038, *p <* 0.001; *post hoc p <* 0.001 – 5° and 15°; *p* = 0.019 − 5° and 10°) (Figure 5B).

### Self-perception

High self-perception scores (≥7) regarding the sense of ownership and agency of the virtual prosthesis by most participants from the beginning of the training. The scores were increased or maintained throughout the protocol, except for two participants, “C” and “D,” who reported a decrease in the sense of agency at the end of the training (Figure 4C).

## Discussion

The results of this study showed that there was induction and enhancement of virtual prosthesis embodiment through training with an EMG-based HMI. We observed that the affective response was immediate, but with training, there was an amplification of this response. These findings, along with the recalibration of the peripersonal space and the increased control capacity with training, showed an improvement in the embodiment over time. The high indices of self-perception declared by the subjects regarding their sense of ownership and agency over the virtual prosthesis also corroborated this.

### Affective measurement

All participants reacted affectively to a threat to the virtual prosthesis, indicated by a significant increase in SCR magnitude, which is a natural physiological reaction to a threat to the subject’s own bodies (Critchley, 2002; Bach et al., 2009; Braithwaite et al., 2015). This response already occurred at the beginning of the protocol, but there was a significant increase at the end, indicating an amplification. Other studies with manipulations of body perception, such as those based on the rubber hand illusion (RHI) paradigm (Botvinick and Cohen, 1998), have also identified an increase in SCR by threatening an external object, indicating embodiment (Armel and Ramachandran, 2003; Ehrsson et al., 2008; Alimardani et al., 2016). In the present study, the increase in SCR magnitude at the beginning of the protocol, suggested that there must have different levels of embodiment, since a few minutes of training to control the virtual prosthesis movements was enough to achieve some embodiment. An explanation for this immediate response may be associated with virtual environment immersion: with no visual feedback from their own body and only a visualization of the virtual body from the first-person perspective, there is a decreased incompatibility between real and virtual body perception in terms of visual, proprioceptive and spatial recognition (Tieri et al., 2015; Burin et al., 2019). This idea is also supported by previous studies that point out that this anatomical congruence between the body itself and an intact virtual limb is sufficient to induce embodiment, even without visuomotor or visuotactile stimulation (Tieri et al., 2015; Fusaro et al., 2016; Pavone et al., 2016).

### Spatial perception measurement

The highest CCE values were obtained when the stimuli were applied on the same side of the body than on the opposite sides at the end of the training, indicating that the visual stimuli applied in the virtual environment were considered close to the real body. In other words, there was a recalibration of the peripersonal space to include the virtual prosthesis (Maravita et al., 2003; Maravita and Iriki, 2004; Van Elk et al., 2013). Although the size of our limbs determines our reach space, the use of tools can alter peripersonal space (Làdavas, 2002; Maravita et al., 2003; Maravita and Iriki, 2004)−a neurocognitive representation produced from the integration of sensory information related to the body itself and the space around it (Holmes and Spence, 2004). Other studies using immersion in virtual environments have also shown that it is possible to extend the peripersonal space to include a tool or virtual limb in an equivalent manner to what is produced in physical environments (Sengül et al., 2012; Shokur et al., 2016). The absence of significant CCE differences at the beginning of the training indicated that the recalibration of the peripersonal space is not immediate and depends on exposure/training, unlike the autonomic/affective response. In the same line of interpretation, Marini et al. (2014), in an experiment using a functional prosthesis, observed that the recalibration of the peripersonal space occurred only after a long period of training. Other studies have also pointed out that the stable recalibration of the peripersonal space depends on the development of skills and prolonged use of a tool or assistive device (Serino et al., 2007; Bassolino et al., 2010).

Considering all this together, our interpretation is that the autonomic/affective response is dependent on the visual and proprioceptive congruence of the real and virtual body experienced through the first-person perspective (Tieri et al., 2015; Fusaro et al., 2016; Pavone et al., 2016). The recalibration of the peripersonal space may be linked to the processing of body perception depending on learning motor skills acquired during the training sessions (Serino et al., 2007; Bassolino et al., 2010; Marini et al., 2014). However, the increase in SCR magnitude and high correlation with the CCE at the end of the protocol indicated that the affective response, although it was immediate, was also strengthened during the learning process, suggesting that the embodiment can have different levels of intensity.

### Motor measurement

An increase in the ability to control the virtual prosthesis was verified by performance analyses during training with the EMG-based HMI, as indicated by the high success rates at all levels of difficulty. Thus, it can be concluded that the participants were able to use visual and vibrotactile feedback for motor planning and execution in the control of the virtual prosthesis movements (De Vignemont, 2011).

The time required for the participants to perform the tasks was longer for the intermediate target angles (30° and 60°) than for the extremities (0° and 90°). For the intermediate angles, the time was even greater when the task required greater precision. The differences in these times can be explained by the level of complexity of the motor control strategies: simpler strategies in the case of extremity angles and more complex strategies for reproducing intermediate target angles, especially in more precise tasks.

This interpretation can be supported by motor control theories based on feedforward and feedback mechanisms (Wolpert et al., 1995; Wolpert and Ghahramani, 2000). In conditions where movement strategies were simpler, motor control occurred largely through feedforward mechanisms from the estimation of sensory consequences using copies of the efferent motor commands. In this way, for the extreme angles, the execution times were shorter because the predicted movements did not require major corrections during the execution. However, during tasks with more complex motor control strategies, those with intermediate target angles and higher precision, motor control occurred mainly through the sensory feedback by comparing predicted and actual movements (Wolpert et al., 1995, 2011; Wolpert and Ghahramani, 2000). In these cases, corrections, and adjustments of the movement in real-time were determinant and explained the longer execution times during these tasks.

### Self-perception

Participants reported high self-perception that the virtual prosthesis was part of their own body and that they could voluntarily control it. This perception remained stable or increased over the course of the training in most cases. Only two participants (C and D) reported a decreased sense of agency at the end of the protocol. However, for both, the score given in the initial evaluation for the agency sense was already the maximum value. Most likely, this result was related to the expectations created by these participants that control would be easier throughout the sessions, which did not occur due to the progressive difficulty increasing imposed during training. Additionally, it is worth noting that this effect did not affect their sense of ownership since both reported an increasing of ownership at the end of the training, which reinforces this interpretation.

The reports of some participants who felt the phantom limb were also interesting and corroborate the self-perception of ownership and agency over the virtual prosthesis: participant C reported that at the end of the protocol he could control the movements of the phantom limb, which he could not do before. Participant E had control over the movements of the phantom limb from the beginning and reported that he used the same strategy to flex the phantom limb to control the knee flexion movement of the virtual prosthesis. Participant F felt the phantom limb in constant flexion and could not move it. However, during the protocol in immersion VR environment, she could actively flex the phantom limb together with the virtual prosthesis movement (“It’s like I have two legs moving”).

Finally, there are two main points of our work that should be highlighted: (a) system and protocol and (b) embodiment investigation.

### System and protocol

The critical difference in the proposed protocol is the combination of components and strategies aimed toward achieving embodiment, in this case, of the virtual prosthesis. These strategies include myoelectric control, immersion in a VR environment and vibrotactile stimulation.

Visual feedback in immersion in a VR environment was chosen based on the results of previous studies that have shown promising effects in a variety of clinical contexts (Bohil et al., 2011; Gumma and Youssef, 2019; Kluger et al., 2019; Qian et al., 2020) and in the induction of the embodiment of a body, limb or virtual object (Slater et al., 2009, 2010; Sengül et al., 2012; Shokur et al., 2016).

Regarding vibrotactile stimulation, we propose using vibrotactile feedback to represent movement (Jones et al., 2009; Shokur et al., 2016). Most current lower limb prostheses do not provide sensory feedback, which makes the user largely dependent on vision to determine the prosthetic limb position and its interaction with the environment. Furthermore, reestablishing proprioceptive sensory information is crucial for the development of embodiment (Proske and Gandevia, 2012; Butler et al., 2017) and improvement of motor control (Riemann and Lephart, 2002; Proske and Gandevia, 2012).

Last, although prosthetic myoelectric control has been widely explored in research and clinical environments (Maruishi et al., 2004; Sebelius et al., 2005; Jackson, 2008; Kluger et al., 2019; Sime, 2019), in general, these studies did not use an immersive virtual environment and focused on control conditions rather than the closed loop between control and feedback, as we propose in this work.

### Embodiment investigation

The embodiment of an external object is a complex concept and experience. Currently, the literature shows an overlap of terms and definitions (De Vignemont, 2011; Makin et al., 2017; Zbinden et al., 2022). Here we based our protocol mostly on the definition provided by De Vignemont (2011); Makin et al. (2017), where there are more practical aspects to be implemented in the therapeutic context: “the ability to process properties of this object at the sensory, motor and/or affective levels in the same way that the properties of one’s own body parts.” This definition is interesting because it inherently brings an ecological and interactive perspective, where the embodiment of an external object can only be achieved if the subjects systematically interact with the environment (including the object itself) through specific sensorimotor criteria.

There are studies that have investigated embodiment through psychophysical tests (Serino et al., 2007; Marini et al., 2014; Shokur et al., 2016), reports and/or electrophysiological activity (Armel and Ramachandran, 2003; Ehrsson et al., 2007, 2008). However, we claim that since different mechanisms are underling and influencing the induction of embodiment (De Vignemont, 2011; Makin et al., 2017; Zbinden et al., 2022), multiple tasks and measurements are required to cover all embodiment dimensions.

This perspective converges with the different aspects and contexts of embodiment reported in the literature, such as quick illusions in experiment of visuotactile congruence (RHI−synchronous tactile stimulation between a rubber hand that is under the visual field and the real hand hidden) (Botvinick and Cohen, 1998) or with visuomotor congruence, in studies involving virtual reality in voluntary control paradigms (Cole et al., 2009; Slater et al., 2009; Ma and Hommel, 2015), and embodiment of assistive technology in the long-term (Serino et al., 2007; Canzoneri et al., 2013; Marini et al., 2014).

Therefore, the association of different factors and mechanisms not only enriches the comprehension of the induction and enhancement of embodiment (in this case, of the virtual prosthesis), but they are necessary (De Vignemont, 2011; Makin et al., 2017; Zbinden et al., 2022).

A limitation of the study is the size and characteristics of the sample. Most of the participants were men and young adults (age range between 18 and 46 years) with traumatic amputations. Studies in people with amputations of other etiologies, ages and in women can clarify what the embodiment process is like under these different conditions.

Future studies with a larger sample and control groups, in addition to randomized clinical trials, are still necessary. Follow-up research is also recommended to obtain a better understanding of whether the modifications are permanent and can be extended to the use of physical prostheses. Nevertheless, these findings show the potential for the use of this system and protocol in the context of rehabilitation of people with amputation in the preprosthetic phase.

## Data availability statement

The raw data supporting the conclusions of this article will be made available by the authors, without undue reservation.

## Ethics statement

The studies involving human participants were reviewed and approved by the Universidade Federal de São Paulo and Hospital Municipal José de Carvalho Florence. The patients/participants provided their written informed consent to participate in this study. Written informed consent was obtained from the individual(s) for the publication of any potentially identifiable images or data included in this article.

## Author contributions

KR contributed to the conception and design of the work, acquisition, analysis, and interpretation of data, and writing and review of the article. JM contributed to the conception of the work, development of programs and devices, and review of the article. DP contributed to the development of devices used in the work and review of the article. RD assisted in analysis of data. TS contributed to the development of devices used in the work. JN did the development of programs used in the work. MN did the conception of the work. EC reviewed the article. JF contributed to the conception and design of the work, analysis and interpretation of data, and writing and review of the article. All authors contributed to the article and approved the submitted version.

## Abbreviations

CCE: crossmodal congruency effect
CCT: crossmodal congruency task
EMG: electromyography
HMI: human-machine interface
RMS: root mean square
SCR: skin conductance response
VR: virtual reality
RHI: rubber hand illusion.
