# Supplementary material for: Embodiment of a virtual prosthesis through training using an EMG-based human-machine interface: Case series
Source: Front Hum Neurosci. 2022 Aug 4;16:870103. doi: 10.3389/fnhum.2022.870103 (PMC9387771; doi:10.3389/fnhum.2022.870103)
Supplement: Supplementary file 1 [file Data_Sheet_1.pdf]

Embodiment of a virtual prosthesis through training using an EMG-based human-machine interface: Case series

Karina Aparecida Rodrigues[1]*, João Vitor da Silva Moreira[1], Daniel José Lins Leal Pinheiro[1], Rodrigo Lantyer Marques Dantas[1], Thaís Cardoso Santos[2], João Luiz Vieira Nepomuceno[2], Maria Angélica Ratier Jajah Nogueira[3], Esper Abrão Cavalheiro[1], Jean Faber[1, 2]

[1]Neuroengineering and Neurocognition Laboratory, Paulista School of Medicine, Department of Neurology and Neurosurgery, Federal University of São Paulo, São Paulo, Brazil
[2] Neuroengineering Laboratory, Institute of Science and Technology, Department of Biomedical Engineering, Federal University of São Paulo, São José dos Campos, Brazil
[3] Center of Rehabilitation Lucy Montoro, São José dos Campos, Brazil

*** Correspondence:**
Corresponding Author
rodrigues.karina@unifesp.br

Supplementary Material

Table 1 - Participants' reports.

Participants	Reports		
	Main complaint	Expectancy	Comments
A	*"I can't drive"*	*"I can use the prosthesis and adapt"*	*"I had a phantom limb sensation, it stopped two months ago"*
B	*"I'm very tired"*	*"I get more independent"*	*"For the first time in 8 years I get to bend my knee"*[*1]
C	*"I can't ride a motorcycle"*	*"Prosthetic fitting"*	*Initial session - "I rarely feel the phantom limb. Final session -" The phantom sensation is rare, sometimes I can control the phantom limb "*
D	*"Use the crutch, both arms are occupied"*	*"Prosthetic fitting"*	*Did not make other reports*
E	*"Difficulty taking a license from work"*	*"Win a prosthesis and use it for everything"*	*"I feel and can control my phantom limb". "When I bend the virtual prosthesis, I do it the same way when I want to bend the phantom knee"*[*2]
F	*"Depend on other people"*	*"Get the prosthesis and do everything"*	*"It's like I have two legs moving"*[*3]
G	*"Back to work"*	*"Get the prosthesis"*	*"I felt the phantom limb only in the first three months after the amputation"*

[*1] This participant had a history of traumatic injury to the lower limb with a series of complications during the attempt to save the limb, culminating in knee arthrodesis and finally in the amputation decision. This whole process up to the surgical amputation procedure lasted approximately 8 years.
[*2] During the first days of training, the patient reported greater difficulty performing knee flexion with the virtual prosthesis.
[*3] The patient reported a sensation of a phantom limb, as if the knee was constantly flexed. She tried to control it, but she could not. During training with the EMG-based HMI and immersion in the VR environment, she reported that when she exerted strength to flex the virtual prosthesis knee, she felt that along with the prosthesis, the phantom limb also flexed.

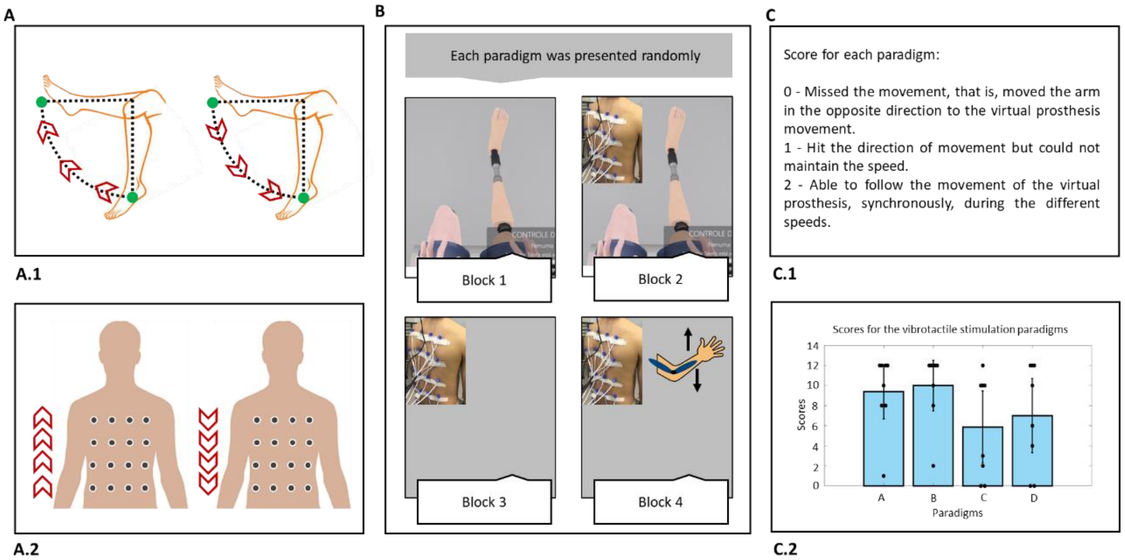

Figure 1 - Identification of the vibrotactile stimulation pattern. A) Possible paradigms: four different combinations (knee extension and upward vibratory stimulation; knee extension and downward vibratory stimulation; knee flexion and downward vibratory stimulation; knee flexion and upward vibratory stimulation) A.1) Movements of the virtual prosthesis: knee extension and flexion. A.2) Application of upward and downward vibrotactile stimuli. B) The presentation sequence of the paradigms was random, and for each one, the following protocol was followed: Block 1 - participants observed the movements of the virtual prosthesis at a speed of 45°/s, without vibrotactile feedback, for 30 s. Block 2 - participants received vibratory stimuli on the back in sync with the avatar movements based on a predefined combination for 60 s. Block 3 - VR glasses were turned off, but the virtual prosthesis continued to move, and the vibrotactile stimulation pattern corresponding to the movement was provided. The participants were instructed to trust vibrotactile stimulation while imagining the virtual prosthesis moving (30 s). Block 4 - VR glasses remained off, but the virtual prosthesis continued to move at different speeds (low = 30°/s; average = 45°/s and high = 180°/s), with corresponding vibrotactile feedback. Participants were instructed to move their arm homolaterally to the amputation at the same speed to reproduce the movements that the prosthesis performed, guided only by vibratory stimuli. For this, a goniometer was attached to the elbow joint to detect movement. In this manner, when the participants wished to represent the extension movement of the virtual prosthesis (leg elevation), they should perform elbow flexion (arm elevation), and the opposite was true for the reverse movement. C) Final score. C.1) Criteria adopted for scoring in each paradigm. C.2) Graph with the final scores (median and CIs) for all participants in each paradigm (A- downward vibration to represent flexion; B- upward vibration to represent extension; C- upward vibration to represent flexion; D- downward vibration to represent extension). The vibrotactile stimulation pattern that generated the highest score in the recognition of each movement performed by the virtual prosthesis was chosen for the training protocol (in the case of a tie, the vibrotactile stimulation pattern that followed the same direction of virtual prosthesis movement was selected).

Table 2 – Details about the virtual reality environment.

Illustration	Description
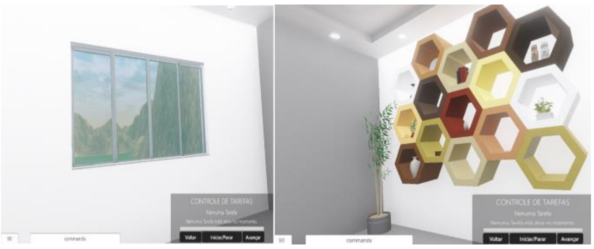	**Virtual room.** Left wall with window and right wall with decorative items.
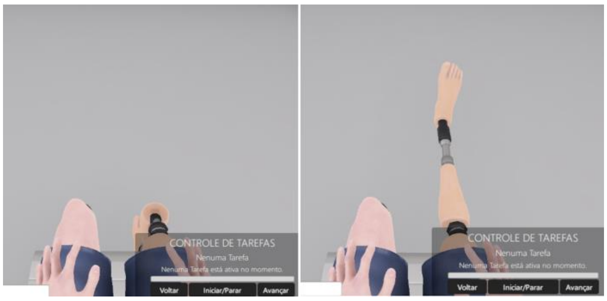	**First-person perspective view.** Motion range limits of 90° flexion and 0° knee extension of the virtual prosthesis.
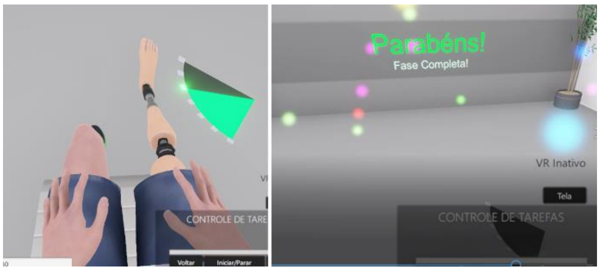	**Training screen with progressive increase in difficulty.** To guide the movements in real time, the participants were presented with a visual clue (semicircular ruler) indicating the position to which they should move the virtual prosthesis. Motivational message “Parabéns (Congratulations)” was showed at the end of each task block.
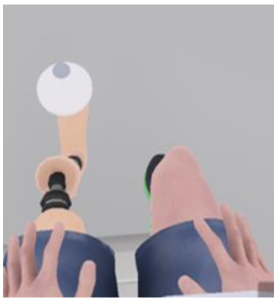	**Screen for affective measurement of embodiment.** Simulation of a threat - a chandelier falling on the virtual prosthesis
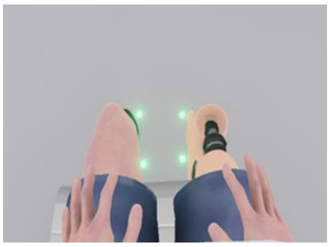	**Screen for spatial perception measurement of the embodiment.** Luminous points (visual distractors) in four different positions: on either side of the hallux or heel.

Table 3 – Details on the application of vibrotactile stimuli on the back.

Illustration	Description
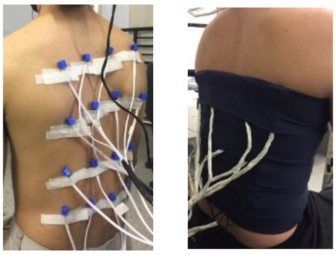	**Vibrotactile stimulation on the back**. Arrangement of 16 vibrotactile actuators in a 4x4 matrix on the back and vibrating actuators conditioned with the aid of fabric strips.<br>The back region was chosen for the application of vibrotactile stimuli, as it has a large area of contact allowing redundant information (protecting the stimulation code). Also, we were thinking about day-use conditions where the feedback on the subject's back would bother much less in terms of functionality. Additionally, a previous study using vibrotactile stimuli to represent military commands, showed a high rate of pattern recognition from stimuli provided in the back region. They also, showed that the individual's ability to identify and perceiving patterns was not affected while performing simultaneous physical or cognitive tasks (Jones et al., 2009).

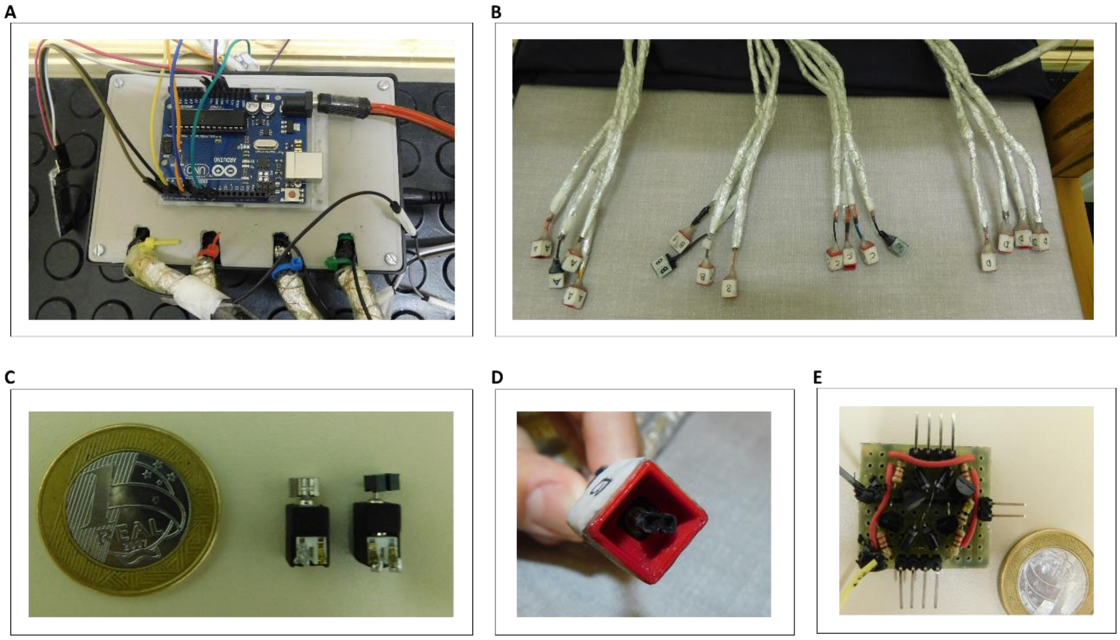

Figure 2 - Vibrotactile stimulation device. A) Final structure of the device. B) Vibrotactile actuators grouped according to each of the stimulation rows. C) Vibrotactile actuators. D) Encapsulated vibrotactile actuators. E) Modular circuit for controlling a row of actuators. The gray wire is the reference, the yellow wire receives the PWM signal that controls the vibration intensity of the actuators. In the pair of pins on the right are inserted the pins positive and negative power supply (3.3V). The lower and upper four pins are the power supply for the four actuators of a module.

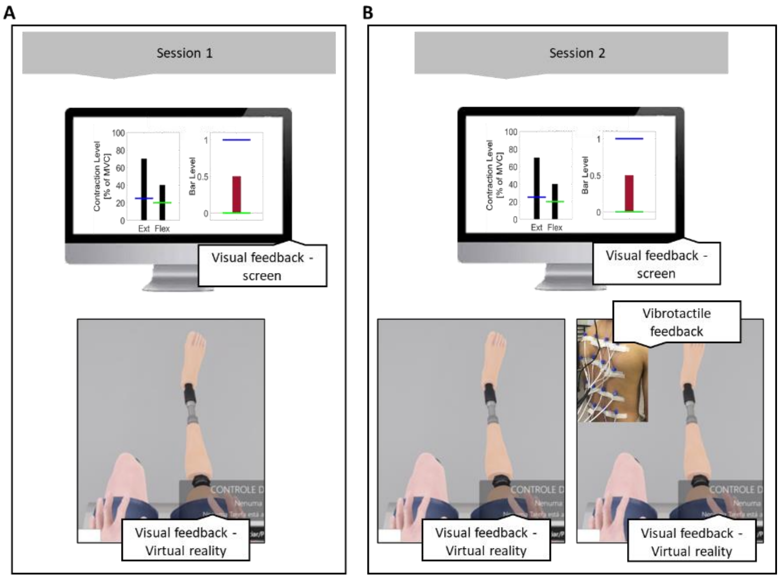

Figure 3 - Familiarization sessions. A) Session 1: Visual feedback on the screen (bars) - four repetitions were performed (in each of them, the participant had to perform four movements up and down the bar). Then, a repetition was performed with visual feedback in the virtual environment, consisting of ten free knee extension and flexion movements of the virtual prosthesis. B) Session 2: Visual feedback on the screen (bars) - three repetitions were performed with five movements up and down the bar. Visual feedback in the virtual environment - three repetitions of free knee extension and flexion movements of the virtual prosthesis. Afterward, the same protocol was repeated with the association of vibratory stimuli on the back to start the association of virtual prosthesis movements. In these sessions, the control parameters consisted of muscle activation above 2 SD of the mean baseline signal and antagonist muscle contraction tolerance of 80%.

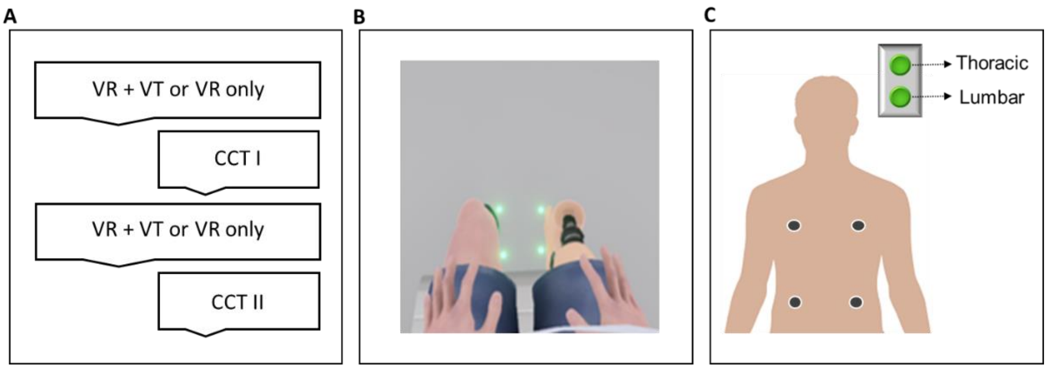

Figure 4 - Spatial perception measurement - crossmodal congruency task (CCT). A) Systematization of task performance. Preview with observation of virtual prosthesis movements (VR only) or association of visualization with corresponding vibrotactile stimuli (VR+VT). After each preview paradigm (presented randomly) the CCT was performed. B) Presentation of visual distractors. C) Arrangement of actuators to provide vibratory stimuli on the back. Buttons for the detection of the response time in the identification of the vibrating stimulus, with options for the thoracic and lumbar locations.
